# Phenotype and natural history in 101 individuals with Pitt-Hopkins syndrome through an internet questionnaire system

**DOI:** 10.1186/s13023-016-0422-2

**Published:** 2016-04-12

**Authors:** Channa F. de Winter, Melanie Baas, Emilia K. Bijlsma, John van Heukelingen, Sue Routledge, Raoul C. M. Hennekam

**Affiliations:** Reinaerde, Organisation for people with intellectual disabilities, Utrecht, The Netherlands; Department of Paediatrics and Translational Genetics, Academic Medical Centre, University of Amsterdam, Meibergdreef 9, 1105AZ Amsterdam, The Netherlands; Department of Clinical Genetics, Leiden University Medical Centre, Leiden, The Netherlands; Pitt-Hopkins Parents Support Group, Leidschendam, The Netherlands; Pitt-Hopkins Parents Support Group UK, London, UK

**Keywords:** Pitt-Hopkins syndrome, Genetic diseases, Intellectual disability, Wiki, E-health, Behaviour, Epilepsy

## Abstract

**Background:**

Pitt-Hopkins syndrome (PTHS; MIM# 610954) is a genetically determined entity mainly caused by mutations in *TransCription Factor 4* (*TCF4*). We have developed a new way to collect information on (ultra-)rare disorders through a web-based database which we call ‘waihonapedia’ (waihona [meaning treasure in Hawaiian] encyclopaedia).

**Methods:**

We have built a waihonapedia system in a collaboration between physicians, social scientists, and parent support groups. The system consists of an initial extensive questionnaire for background cross-sectional data, and subsequent follow-up using small questionnaires, with a particular focus on behavioural aspects. The system was built to be used through the internet, ensuring a secure environment, respecting privacy for participants, and acting automated to allow for low costs and limiting human mistakes in data handling. Recruitment of participants is through the patient support groups. In addition, as a sub-study, we used the data from the waihonapedia system to compare the two proposed diagnostic classification systems for PTHS.

**Results:**

We present here the results of the initial, cross-sectional questionnaire in which early development, physical health, cognition and behaviour are interrogated, and to which modules specific for PTHS were added on epilepsy and breathing patterns. We describe 101 individuals with a molecularly confirmed diagnosis of PTHS.

Comparison of the two classification systems aimed at helping the clinical diagnosis was performed in 47 of the present PTHS individuals, with disappointing results for both. Internationally accepted clinical diagnostic criteria are needed.

**Conclusion:**

The present cross-sectional data on the natural history of PTHS have yielded useful information which will further increase when follow-up data will be added. No doubt this will improve both care and research.

## Background

Pitt-Hopkins syndrome (PTHS; MIM# 610954) is an infrequently reported entity, first described in 1978 in two unrelated individuals [1], and characterized by an intellectual disability, abnormal breathing patterns, and a specific facial gestalt, including deep-set eyes, a broad nasal base and a wide mouth with a tented upper lip [2–6]. Reliable figures for prevalence are not available. The gene *TransCription Factor 4* (*TCF4*; MIM# 602272), located at 18q21 and encoding a class I basic helix-loop-helix transcription factor (bHLH), has been found to be the main disease causing gene [7–9]. A large number of different TCF4 mutations and deletions have been described, with approximately 40 % point mutations, 30 % small deletions/insertions, and 30 % deletions [3]. In early human development TCF4 is highly expressed in the central and enteric nervous system, the sclerotome, the peribronchial and kidney mesenchyme, and the genital bud [10]. Tissue specificity of TCF4 expression and its interaction with other bHLH proteins are likely to explain the intellectual and developmental disabilities, unusual breathing patterns and epilepsy in PTHS individuals, as well as MRI brain abnormalities such as underdevelopment of the corpus callosum and hippocampus, posterior fossa abnormalities and ventricular dilatation [3, 4, 10, 11]. It has been suggested that missense mutations are associated with higher seizure activity [5], but others did not detect a genotype-phenotype correlation [3]. Additional manifestations of PTHS are ophthalmologic signs (strabismus, myopia, astigmatism), epilepsy, constipation, gastro-oesophageal reflux, scoliosis, ataxic gait, hypotonia, and cryptorchidism and other genital malformations [3, 4]. Reports on recurrent infections are ambiguous [3, 12]. Since PTHS individuals were found to have low IgM levels and TCF4 targets several immunoglobulin enhancer sites, it has been suggested that long-term follow-up is needed to evaluate susceptibility to infections, autoimmune disorders and tumours [10]. Individuals with PTHS typically show a smiling appearance, but may also have an anxious or agitated disposition, and they have frequently stereotypic movements [3, 4]. Based on cross-sectional samples, two different systematics have been suggested to help determining whether molecular analysis of *TCF4* is indicated and establishing a clinical diagnosis (Table 1) [3, 13].

**Table 1:** Presence of Published Criteria Helping Establishing the Diagnosis in Present Cohort of PTHS individuals (items occurring in > 75 % are indicated in bold)

Feature	Marangi score	Score cohort (%)	Whalen score
Severe intellectual disability	2	47/47 (**100** %)	
Absent speech (<5words)	2	32/47 (68 %)	2
Severe speech impairment^a^	1	15/15 (**100** %)	
Growth at birth normal	1	29/47 (62 %)	
Overgrowth		1/47 (2 %)	−1
Postnatal decrease of OFC	1	7/27 (26 %)	
OFC < −3SD		0/47 (0 %)	−2
Epilepsy/EEG abnormalities	1	17/47 (36 %)	
Ataxia/motor incoordination	1	26/47 (55 %)	1
Walking >3 years/severe motor delay <3 years		44/47 (**94** %)	2
Hyperbreathing/apnoea	1	19/47 (40 %)	1
Constipation	1	40/47 (**85** %)	
Abnormal brain MRI^b^	1	3/12 (25 %)	
Eye anomalies	1^c^	25/47 (53 %)	
Eye anomalies		21/47 (44 %)	1^d^
Typical PTHS facial features^e^	4	24/47 (51 %)	
Partial typical PTHS facial features	2	23/47 (49 %)	
Deeply set eyes		23/41 (56 %)	1
Protrusion mid/lower face		36/47 (**77** %)	1
Broad nasal bridge *or* convex nasal ridge^f^		44/45 (**98** %)	1
High nasal bridge^f^		30/44 (68 %)	1
Flared nostrils		27/47 (57 %)	1
Large mouth		37/47 (**79** %)	1
Tented vermillion of upper lip^f^		20/47 (43 %)	1
Everted vermillion of lower lip^f^		37/46 (**80** %)	1
Visceral malformations		1/47 (2 %)	−1
Stereotypy head/hands		22/47 (47 %)	2
Loss of purposeful hand skills		0/47 (0 %)	−1
Hypotonia		27/47 (57 %)	1
Smiling appearance		24/47 (51 %)	1
Anxiety/agitation		16/47 (34 %)	1
Check for *TCF4* mutation indicated	If total score ≥10	29/47 (62 %)	
		3/42 (7 %)	Patients ≥3 years: If total score ≥ 16
		5/5 (100 %)	Patients <3 years: If total score 10–15
Diagnostic	If total score ≥13	4/47 (9 %)	-

^a^More than 10 words; 2–3 word sentences

^b^Small callosal body; wide ventricles; thin hindbrain

^c^Strabismus, myopia, astigmatism

^d^Strabismus

^e^Bitemporal narrowing, square forehead, deeply set eyes, hypotelorism, upslanted palpebral fissures, arched or thin eyebrows, broad nasal bridge, pointed nasal tip, flaring nostrils, short philtrum, wide mouth, tented vermillion of upper lip, thick lower vermillion^f^

^f^ Terminology changed to comply with international terminology “Elements of Morphology”

Longitudinal data on series of individuals with PTHS are lacking. In general, the main factor hindering the collection of data on the natural history of rare disorders is formed by the small number of affected people within individual countries and limited number of clinicians and researchers working on each rare entity. But patients and their families know the syndromes best. Families are the continuous factor in the patients’ lives, and know most about their development, behaviour and health problems. This urges for the collection of longitudinal information from large international groups within a collaboration between clinicians, researchers and families. Thus, support groups should play a major role, as typically they are organised internationally, have often digitally communication structures and invariably are very willing to contribute as gathering the information is also a main goal for them [14].

We have recently built an internet database which allows to gather longitudinal data on rare disorders, which we called ‘waihonapedia’ (waihona [meaning treasure in Hawaiian] encyclopaedia) [14]. The waihonapedias for various rare disorders will offer unprecedented and extremely valuable information on the natural history of disorders. Prerequisites for a reliable waihonapedia are a firm diagnosis, a secure database and guaranteed privacy. Participants of each waihonapedia have their diagnosis confirmed by biochemical, metabolic or molecular testing, or, if not possible, diagnoses are assessed by medical advisors of support groups with outstanding experience in the disorder. Individuals with (ultra-)rare disorders are easily recognisable, especially in combination with only limited personal data like age, gender and country of origin, so safe data transmission is an absolute prerequisite to guarantee privacy for participants and to obtain permission of both medical ethics committees and patient groups to collect data.

Here we report on the information from the baseline questionnaire of the PTHS waihonapedia. This includes data on development, physical health and cognition, and possible genotype-phenotype correlations of the first 101 individuals with PTHS in the waihonapedia database. In addition we describe the results of evaluation of the two classification systems of clinical diagnosis criteria within part of the cohort.

## Methods

### Design

This is the cross-sectional baseline measurement of an international long-term follow-up study into the natural history of PTHS. The study has been approved by the medical ethics committee of the Academic Medical Centre in Amsterdam (W15_180#15.0217). The study adheres to the Declaration of Helsinki for research involving human subjects [15].

### Participants

Participants were recruited through the Dutch and international Pitt-Hopkins Support Groups. Eligible for the study were all individuals with a molecularly confirmed diagnosis of PTHS. Support groups promoted the study within their digital infrastructures (e.g. sites, social media, newsletters), and contacted parents or caregivers of eligible participants individually. As a result, parents/caregivers emailed us to sign up for the questionnaire, or, if the parents/caregivers consented, support groups forwarded contact data to the research group. The research group invited each family formally to the study, with written information, and subsequently a personal invitation was forwarded allowing the families to fill out the online questionnaire.

### Data collection

A questionnaire in lay language has been designed based on published data on PTHS and general information about basic development by 2 parents (JvH, SR), a psychologist (MB), and a paediatrician-clinical geneticist (RCMH). Subsequently, a pilot questionnaire was evaluated for clarity and face validity by 5 families, which led to somewhat adapted definitive questionnaire. It comprises 146 questions with main themes: general information (date of birth, gender, country, weight, height, date of completion of the questionnaire), the diagnosis (exact cause of PTHS, when and who diagnosed it), growth, development (cognition, milestones, schooling, formal testing), natural history (pregnancy, delivery, neonatal period, feeding, toilet habits, sleeping, (unusual) movements, epilepsy, problems with internal organs, infections), breathing patterns, appearance (face, hair, teeth, skin, joints, hands, feet), sensory organs (vision, smell, hearing), other diseases and admittance in hospital, and any additional studies (electro-encephalography, brain imaging). The questionnaire was available in English and Dutch. Although not mandatory we invited families to forward clinical pictures of the child with PTHS, to allow for scoring of facial and limb characteristics by the research group. Participants could fill out the questionnaire at once, but could also pause, as answers remain stored, and continue at a later moment. Participants could always correct previous answers until the answering was made definitive. The questionnaires themselves were coded, with as single key a number that could be linked to personal (contact) data. The key is stored in a secure environment in the Academic Medical Centre in Amsterdam. Data were collected between July 1, 2013 and July, 1 2015.

For the evaluation of the systems helping to diagnosis PTHS clinically (Table 1) we used the clinical pictures that were available of 47 participants. Two clinical geneticists with exceptional experience in PTHS (EKB; RCMH) scored the PTHS participants independently based on the information in the questionnaire and the pictures.

### Statistical analysis

Data were converted from the online questionnaire into an anonymous SPSS file. Data were analysed using IBM SPSS Statistics version 21.0 (Chicago, Il). Descriptive statistics were used to provide information on prevalence and details on the various participant characteristics. Chi-square analysis was performed to explore genotype-phenotype correlations.

## Results

### Participants

We invited 139 families to participate. Of these, 101 families (72.7 %) completed the questionnaire. Families indicated it took them 1 up to 5 hours to complete the entire questionnaire. 54.5 % of the participating PTHS individuals were men, the mean age was 9 years (median age 8; range 0–32 years). Most participants came from the USA (*n* = 36), the Netherlands (*n* = 17), and the UK (*n* = 16) but also participants from 16 other countries contacted the research group to sign up for the study. The general data on participants and diagnosis are shown in Table 2. Diagnostic processes differed, with some receiving the diagnosis shortly after birth and others years later, the oldest being 20 years when the correct diagnosis was made.

**Table 2:** Descriptives of the 101 Individuals with PTHS

		Percent
Male		54.5
Female		45.5
Age	0–5 years	40.6
	6–10 year	24.8
	11–15 years	17.8
	16–20 year	6.9
	21–25 years	7.9
	26–30 year	1.0
	31–35 years	1.0
First suspicion something was wrong	During pregnancy	3.1 %
	Birth-6 months	56.3 %
	>6 months-1 year	38.5 %
	>1 year	2.1 %
Age at diagnosis		Mean 7.1 year, range: 3 weeks–27 years
Cause of PTHS	*TCF4* deletion	41.1 %
	*TCF4* mutation	51.1 %
	*NRXN1* mutation	2.2 %
	Parent not sure but abnormality present	5.6 %
Who made the diagnosis	Geneticist	86.6 %
	Paediatrician	7.2 %
	Neurologist	5.2 %
	Other	1.0 %

### Pregnancy, neonatal period, and development

Five people noted intra-uterine growth retardation and two mentioned limited intra-uterine movements (Table 3). Most parents first suspected that something was wrong because milestones were not reached. Other signals picked up as first signs by parents were failure to thrive, gastrointestinal problems (such as reflux, feeding problems, vomiting, constipation), and other symptoms, such as cyanosis during crying, breathing problems, seizures and abnormal muscle tone. Some parents noted an unusual facial appearance. Development was generally delayed. Details about milestones are available in Table 4.

**Table 3:** Data on Pregnancy and Neonatal Period in 101 PTHS individuals

Pregnancy	Hypertension	10.5 %	
	Diabetes	3.1 %	
	Other	17.2 %	(2x hypothyroidism, 1x low iron level, 7x infections, 1x celiac disease, 1x colitis ulcerosa, 2x placenta praevia)
	Delivery <37 weeks	9.6 %	
Weight at birth	Low birth weight for duration of pregnancy (<−2SD)	6.8 %	
	Mean birth weight of children born at term (pregnancy ≥ 37 weeks)	3173 g	
Head circumference at birth	Boys (*n* = 19)	Mean 33.6 cm, SD 1.6	Range 30–37.0 cm
	Girls (*n* = 15)	Mean 32.9 cm, SD 2.6	Range 26–37.5 cm
Start	Apgar score at 1 min	≤6	8.4 %
		7–10	68.7 %
		Not sure	22.9 %
	Apgar score at 5 min	6	2.5 %
		7–10	75.3 %
		Not sure	22.2 %

**Table 4:** Developmental Milestones per Age Category in 101 PTHS Individuals

Current skills		Participants in age categories having acquired skill (age in years)						
		0–2 years (*n* = 16)	3–5 years (*n* = 22)	6–10 year (*n* = 23)	11–15 years (*n* = 16)	16–20 year (*n* = 4)	21–25 years (*n* = 8)	26 years + (*n* = 2)
Laughing		86.7 %	100 %	100 %	100 %	100 %	85.7 %	100 %
Making noises		93.8 %	100 %	100 %	93.3 %	66.7 %	100 %	100 %
Keeping head up		87.5 %	95.2 %	100 %	93.3 %	100 %	100 %	100 %
Grasping objects		87.5 %	90.5 %	90.5 %	86.7 %	66.7 %	71.4 %	100 %
Turning from back to belly		81.3 %	90.5 %	90.5 %	86.7 %	100 %	85.7 %	100 %
Sitting unaided		56.3 %	90.9 %	95.5 %	100 %	100 %	85.7 %	100 %
Crawling		12.5 %	68.2 %	59.1 %	46.7 %	33.3 %	33.3 %	0.0 %
Standing unaided		12.5 %	28.6 %	77.3 %	73.3 %	66.7 %	71.4 %	100 %
Walking with aid		25.0 %	66.7 %	85.7 %	80.0 %	66.7 %	85.7 %	100 %
Walking unaided		6.3 %	31.8 %	76.2 %	78.6 %	33.3 %	71.4 %	100 %
Eating unaided		75.0 %	72.7 %	100 %	93.3 %	100 %	83.3 %	100 %
Eating with aid		31.3 %	50.0 %	57.1 %	37.5 %	66.7 %	57.1 %	100 %
Dressing unaided		0.0 %	0.0 %	4.8 %	0.0 %	0.0 %	14.3 %	0.0 %
Speaking single words		6.3 %	18.2 %	54.5 %	31.3 %	25.0 %	42.9 %	100 %
Speaking whole sentences		0.0 %	0.0 %	9.1 %	0.0 %	0.0 %	14.3 %	0.0 %
Potty trained urine		6.3 %	9.1 %	17.4 %	18.8 %	0.0 %	14.3 %	0.0 %
Potty trained poo		12.5 %	27.3 %	26.1 %	37.5 %	25.0 %	12.5 %	0.0 %
Previous and current problems								
Feeding Problems	Previously	31.3 %	50.0 %	22.7 %	37.5 %	0.0 %	28.6 %	50.0 %
	Currently	31.3 %	4.5 %	4.5 %	0.0 %	0.0 %	0.0 %	0.0 %
Drinking problems	Previously	6.3 %	22.7 %	27.3 %	12.5 %	0.0 %	12.5 %	0.0 %
	Currently	31.3 %	13.6 %	18.2 %	12.5 %	0.0 %	25.0 %	0.0 %
Solid food problems	Previously	6.3 %	22.7 %	13.6 %	37.5 %	0.0 %	14.3 %	0.0 %
	Currently	37.5 %	31.8 %	13.6 %	31.3 %	25.0 %	0.0 %	0.0 %

### Physical health

**Fig. 1:**
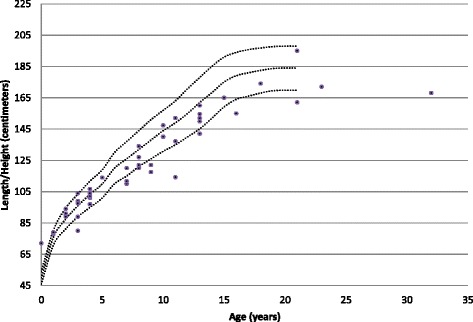
Boys length/height according to age

**Fig. 2:**
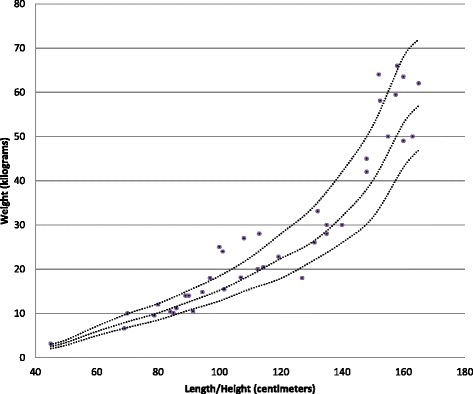
Boys weight according to length/height

**Fig. 3:**
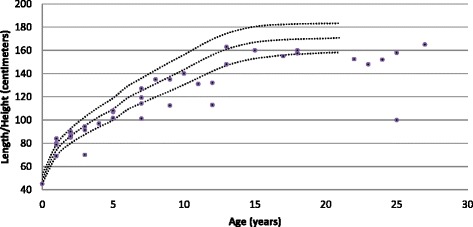
Girls length/height according to age

**Fig. 4:**
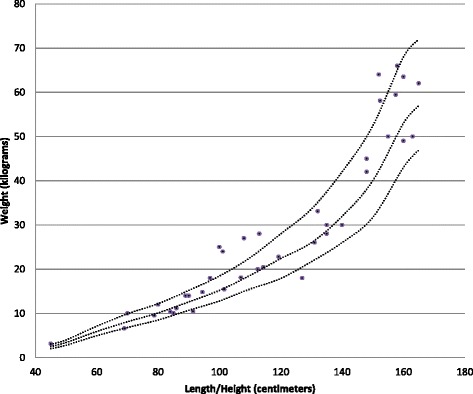
Girls weight according to length/height

**Fig. 5:**
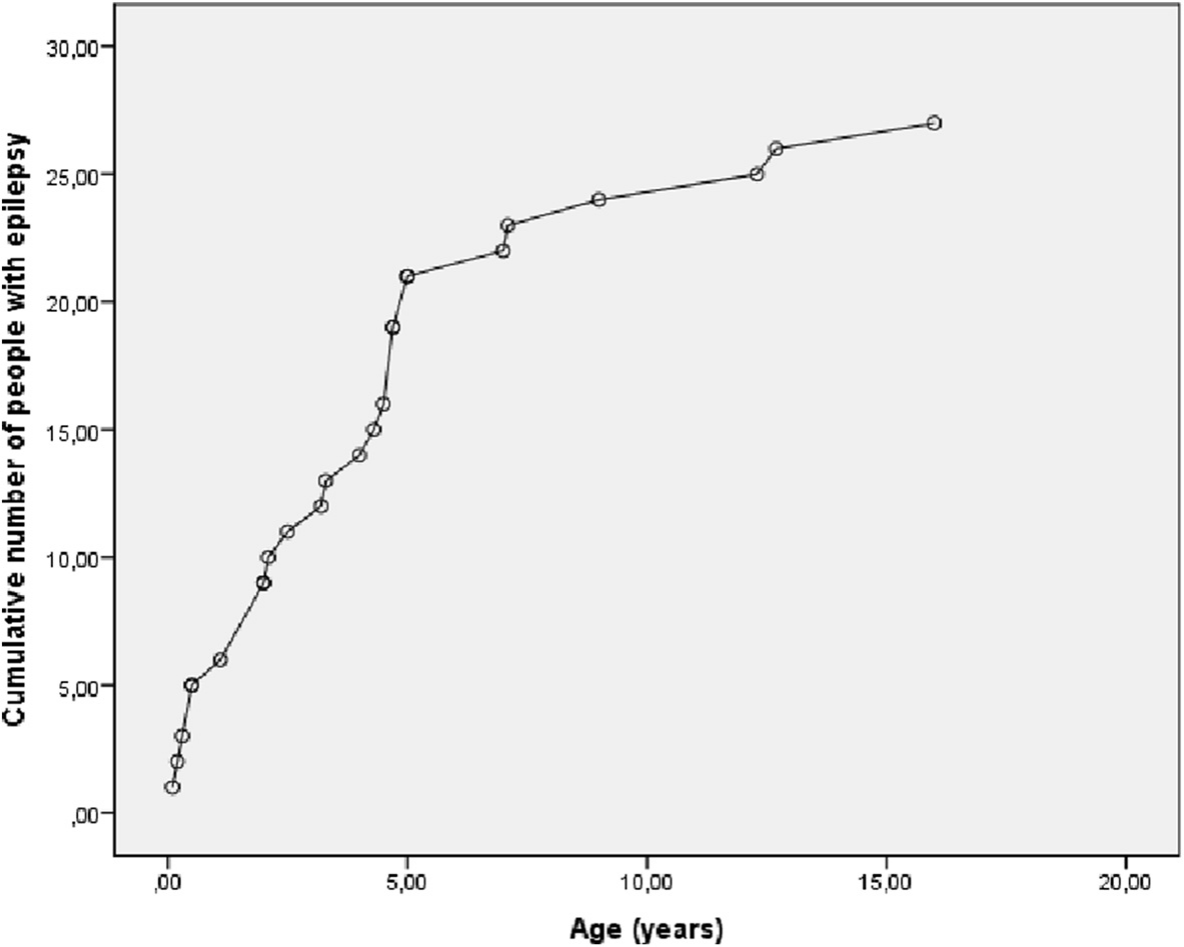
Cumulative number of individuals with Pitt-Hopkins syndrome who developed epilepsy according to age

Main physical health parameters are shown in Table 5. Weight in relation to height, and height in relation to age are shown in Figs. 1, 2, 3 and 4. The gradual development with age of epilepsy is shown in Fig. 5. Of the 38 participants with epilepsy, 23.7 % became seizure free at a mean age of 6.4 years. Most (51.4 %) had seizures that included involuntary motor activity or tonic-clonic seizures, often in combination with absences. 16.2 % had absences only, for the remaining participants with epilepsy, parents were not sure about the type of seizures. An increase of the epilepsy was often noted during fever. Overbreathing or apnoea’s shortly before a seizure were also mentioned in 7 participants. 24 participants were on anti-epileptic drugs, with various anti-epileptics used, mostly valproate, levetiracetam, lamitrigine and carbamazepine, or combinations of these. There was no specific medication that was more successful in decreasing seizures than other medications. Seizures remained therapy-resistant in some participants, but in most therapy was successful. EEGs showed no epileptic activity in 52.2 % of the cases (some were already on anti-epileptic drugs or received sedative medication). In all others epileptic activity was found. Lennox-Gastaut syndrome was mentioned once. Neuro-imaging was performed in 34 of the participants with at least one epileptic seizure during their life, and showed abnormalities in 2/3rd of them. Findings were a small or absent callosal body (*n* = 4), an arachnoid cyst which caused hydrocephaly and needed surgical correction, another small arachnoid cyst without clinical consequences, a hydrocephaly needing shunting, and a diffuse reduction in white matter volume and symmetric bilateral increase in T2 signal, involving the globus pallidus.

**Table 5:** Physical Health Problems in 101 PTHS Individuals

Age	0–2 years (*n* = 16)	3–5 years (*n* = 22)	6–10 year (*n* = 23)	11–15 years (*n* = 16)	16–20 year (*n* = 4)	21–25 years (*n* = 8)	26 years + (*n* = 2)
Epilepsy							
History of seizures	31.3 %	18.2 %	17.4 %	62.5 %	0 %	62.5 %	50.0 %
Currently seizures (out of those with a positive history)	16.7 %	100 %	44.4 %	45.5 %	0 %	60.0 %	50.0 %
Breathing							
History of breathing anomalies	20.0 %	22.7 %	21.7 %	68.8 %	100 %	50.0 %	100 %
Currently breathing anomalies (out of those with a positive history)	50.0 %	80.0 %	50.0 %	70.0 %	100 %	75.0 %	100 %
Gastrointestinal problems							
Gastro-oesophageal reflux	56.3 %	42.9 %	33.3 %	28.6 %	25.0 %	14.3 %	50.0 %
Constipation	73.3 %	81.0 %	86.4 %	86.7 %	66.7 %	62.5 %	100 %
All ages							
(Excessive) burping	28.7 %						
Other gut problems	2.4 %						
Vision							
Near-sighted	31.7 %						
Far sighted	21.7 %						
Strabismus	44.7 %						
Nystagmus	19.0 %						
Tear duct blockage	11.8 %						
Hearing							
Examined	97.7 %						
Impaired	10.3 %						
Ability to smell							
Hypersensitive to smell	2.4 %						
(Seemingly) inability to smell	1.2 %						
Mouth/tooth							
Caries	8.3 %						
Excessive drooling	81.4 %						
Other intra-oral problems	41.2 %						
Urogenital problems							
Retractile testicles (*n* = 48)	6.3 %						
Uni-/bilateral cryptorchidism	33.3 %						
Surgical correction cryptorchidism	50.0 %						
Other urogenital problems	17.4 %						
Muscles/joints							
Hypotonia	75.9 %						
Hypertonia	6.9 %						
Dislocated joints	1.1 %						
Limited mobility fingers/wrists	27.9 %						
Infections							
Frequent recurrent infections	35.2 %

**Fig. 6:**
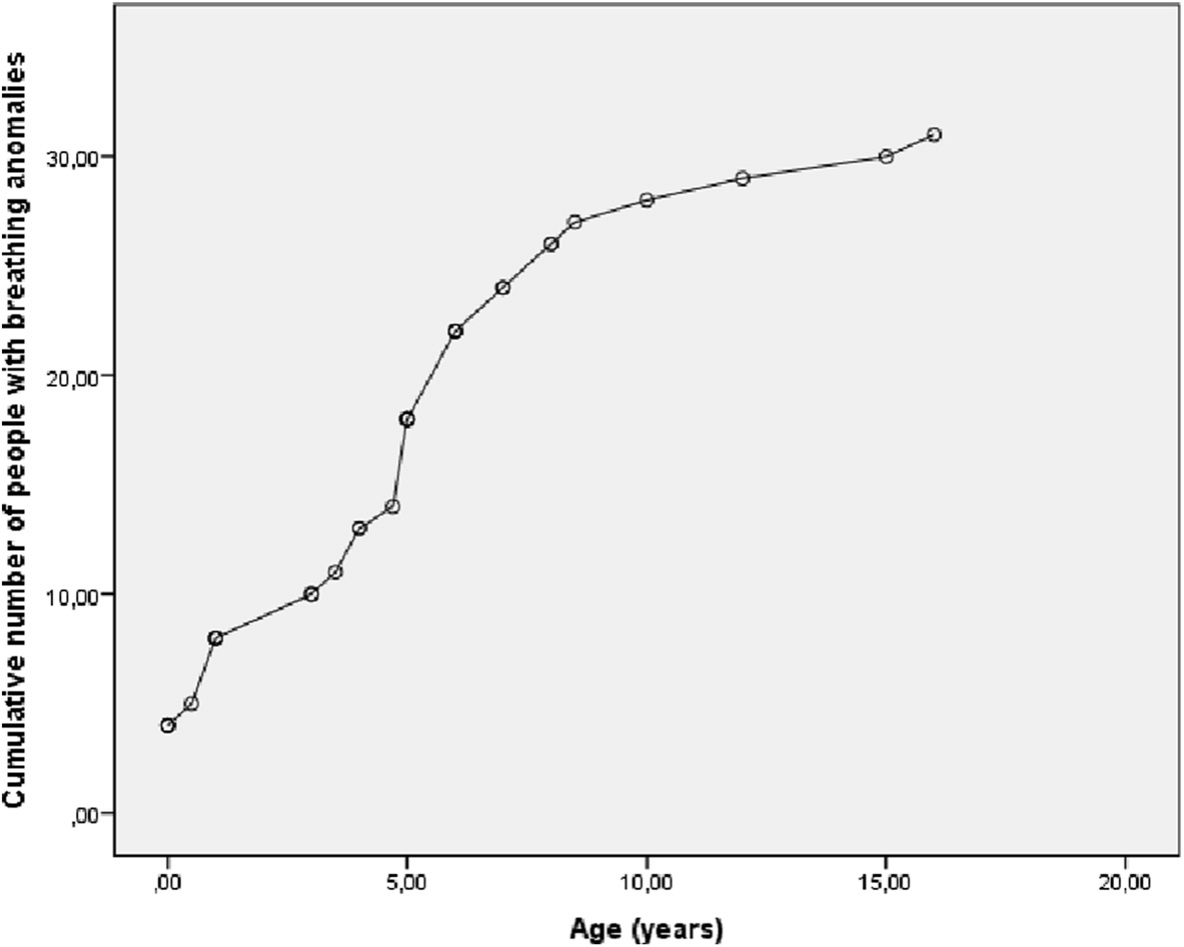
Cumulative number of individuals with Pitt-Hopkins syndrome who developed breathing anomalies according to age

The development of breathing anomalies in time is shown in Fig. 6. Typically breathing anomalies increased with time, but of the 34 PTHS individuals with breathing anomalies 3 individuals subsequently stopped to have breathing problems (mean age of stopping 4.3 years). In one patient improvement of hyperbreathing pattern coincided with cardiac surgery. Breathing anomalies consisted usually of periods of rapid, sometimes irregular breathing followed by breath holding. Sometimes this cycle repeated itself several times one after another. Rapid breathing episodes could be preceded by excitement, stress or anxiety, but in others there was no apparent reason and the participant remained comfortable during both hyperbreathing and apnoea. Hyperbreathing may lead to a swollen abdomen in 1/5^th^of the PTHS individuals and a quarter turned pale with blue lips during apnoea’s, some fainted. One participant had obstructive sleep apnoea’s.

Gastrointestinal problems were common, especially constipation (Table 5). Gastro-oesophageal reflux was present in 37.6 % of the participants, and decreased with age. Other gut problems were pyloric stenosis and malrotation. Toothing and shedding of the milk teeth usually occurred at normal age. Other intra-oral problems noted were ulcers (in 3 participants), fused teeth (in 3 participants), cleft lip and palate (once), a high palate (once) and widely spaced teeth (twice). Open mouth behaviour was mentioned in a few cases, but excessive drooling was mentioned frequently (in 81 %).

Infectious disease included recurrent otitis, tonsillitis, frequent pneumonias, and once an endocarditis. Immunological disturbances were reported twice: a low IgA level and a low IgG level. There was one boy with recurrent bladder infections. Genital problems were a buried penis, small penis, vaginal aplasia (which may have been accompanied by absent uterus and ovaries, but this remains uncertain) and underdeveloped internal genitalia.

Vision was frequently disturbed needing glasses, and hearing was disturbed only infrequently (Table 5). In addition to refraction errors, also strabismus, nystagmus, astigmatism, disturbed depth vision and cortical visual impairment occurred.

### Behaviour

Behavioural characteristics are summarized in Table 6. Behaviour during feeding includes gagging, choking and not chewing properly. Some refuse food, or have very strict rituals during feeding, but in general many are described as excellent eaters. Sleeping patterns are described in Table 6. Many parents mentioned that their child sleeps excellently.

**Table 6:** Behavioural Characteristics in 101 PTHS Individuals

Behaviour		Percent
Unusual behaviour during feeding		40.4 %
Sleep problems	No longer periods of sleep/frequently awake at night	4.4 %
	Other sleep problems	4.4 %
Problems falling asleep	Previously	12.1 %
	Currently	17.6 %
Problems sleeping the whole night through	Previously	13.6 %
	Currently	33.0 %
Night terrors	Previously	6.6 %
	Currently	5.5 %
Hand biting	Previously	11.4 %
	Currently	58.0 %
	Mean age of start	4.2 years (range 0–23 years)
Teeth grinding	Previously	28.9 %
	Currently	46.7 %
	Mean age of start	2.8 years (range 0–12 years)
Clapping/flashing/hand washing	Previously	9.0 %
	Currently	80.9 %
	Mean age of start	1.7 years (range 0–15 years)
Repetitive movements	Previously	4.4 %
	Currently	66.7 %
	Mean age of start	3.3 years (range 0.2–12 years)

(Unusual) repetitive movements are described frequently, such as head shaking, head banging, body rocking and flapping, washing, clapping with arms and hands. Flicking fingers is mentioned once. Hand washing movements as in Rett syndrome are not uncommon, although clapping and flapping of the hands occur mostly. Other unusual behaviour is rubbing the toes together, hair pulling, temper tantrums, inappropriate laughing, hyperextending body or legs, and throwing or banging or kicking on objects. Also pinching, pressing and hitting oneself are mentioned.

Information on formal cognitive testing was available from 21 participants. They were tested between 1.5 and 32 years of age (mean 8.8 years), and the range of developmental age was between 9 and 36 months (mean 14.5 months).

### Genotype-phenotype correlations

No genotype-phenotype correlations were found for epilepsy (chi-square = 4.98, *p* = .55) and breathing anomalies (chi-square = 1.11, *p* = .98). This indicates that the prevalence of epilepsy and breathing anomalies was distributed equally over the groups with a *TCF4* deletion, *TCF4* mutation and *NRXN1* mutation.

### Comparison of diagnostic classification systems

The scoring of the individual facial signs was performed by two of us (EB; RCMH), using the available clinical pictures. The quality of the pictures was not always sufficient to score all signs; if in doubt a sign was not scored. The correlation of the two scorers for the specific signs was good: they scored in total 678 signs of which they concurred in 596 (88 %).

The total set of criteria defined by Whalen^3^ as sufficient to indicate molecular analysis of *TCF4* was met by 8 of the 47 PTHS individuals (17 %), and by the set of criteria defined by Marangi^4^ indicated that 29 of the 47 PTHS individuals (62 %) should have been checked molecularly. Marangi and co-authors indicated that a score of 13 or higher was diagnostic for PTHS: this was met by 4 of the present 47 individuals (9 %). As stated above all participants of the present study had a molecularly confirmed diagnosis of PTHS.

## Discussion

We have initiated a project which should lead to careful, extensive data of a large series of individuals with PTHS and several other genetically determined disorders creating and using the web-based questionnaire system waihonapedia. The present report is the first report of the series of PTHS individuals, describing the cross-sectional data obtained at starting the project. Follow-up as part of the waihonapedia system will allow to follow the present individuals in time and obtain better insight in the natural history. We aim at expanding the number of PTHS participants as our aim is to follow a cohort of at least 250 individuals. Furthermore the data on individuals with various entities in which we are using or will use the waihonapedia system (PTHS; Cornelia de Lange syndrome; Rubinstein-Taybi syndrome; Marshall-Smith syndrome; Beckwith-Wiedemann syndrome; Nicolaides-Baraitser syndrome; tuberous sclerosis; rare chromosome anomalies and likely several others) will also allow comparison of data across syndromes, which will allow to determine symptoms and complications that are truly typical for each syndrome. The waihonapedia system is safe, has arrangements to secure privacy of participants, and is fully automated and cheap [14], which are all prerequisites for success.

The major characteristics on development, health and behaviour of a relatively large cohort of PTHS individuals is provided here in Tables 2, 3, 4, 5 and 6. We confirmed that the primary features of PTHS of the total group of participants are intellectual disability (100 %), epilepsy (32 %) and respiratory anomalies (38 %). In most cases overbreathing increased with age, but in a few cases it diminished over time. There was no statistically significant genotype-phenotype correlation for epilepsy and breathing anomalies, similar to what has been reported before [3]. Reflux (38 %) and constipation (80 %) are also common in the total group.

As a sub-study we combined the data from the present questionnaire with evaluation of the facial morphology of those participants that uploaded pictures (half of the present cohort), to evaluate whether the two sets of criteria that are used in practice [3, 13], yielded different results. The results indicated that in general scoring of facial signs by scorers experienced in a particular entity using pictures submitted by families, shows a highly reliable correlation (scoring 88 % in agreement). The set of criteria as defined by Marangi and co-workers^4^ to be sufficient to establish a clinical diagnosis was met in 8.5 %. A score sufficient to warrant molecular studies of TCF4 was met by 62 % (Marangi criteria) or 17 % (Whalen criteria), although a molecularly confirmed diagnosis was present in all participants. The Whalen criteria contained several items which decreased the score if present, such as overgrowth, visceral malformations and the loss of purposeful hand skills. Likely this was added to discern PTHS from other, similar entities such as Rett syndrome. Although useful in clinical practise such signs or symptoms will be hardly present and will decrease the score markedly. In our opinion it is not useful to add such items in a set of criteria: positively scoring items, especially those that are not frequently found in related entities, will be the most valuable. The facial signs as used by Whalen and co-authors scored the characteristics in the present cohort better than the facial signs as mentioned by Marangi and co-authors: the typical PTHS facial features, defined as the presence of more than 50 % of the facial signs mentioned in the criteria was present in 32/47 using the Whalen score and 24/47 using the Marangi score.

While evaluating this relatively large number of PTHS individuals in a short time both present scorers independently noticed that there was often an asymmetry in the upslant of the palpebral fissures and indeed of the upper 1/3rd of the face in general (present in 11/47). In addition both noticed that the medial 2/3rd of the eyebrows can be broad but it is especially the lateral 1/3rd that is narrow and pencilled. We conclude that the present sets of systematics helping in determining in whom to evaluate the presence of an TCF4 mutation may be helpful but are not yet sufficiently precise to be used as diagnostic criteria. Well-functioning, internationally accepted criteria are therefore missing, while these are mandatory for optimal diagnostics and management, and such initiative is urgently needed.

One may argue that diagnostic criteria are no longer useful or needed as targeted whole exome or whole genome sequencing will rapidly be used as a first diagnostic tool in individuals with intellectual disability. However, the increasing yield of mutations in genes in individuals who do not resemble the entity/entities known to be caused by these genes, will ask for a more detailed evaluation of the phenotype in order to assess causality of the variant. Therefore detailed phenotyping is more needed than ever [16].

Strengths of the present study are the relative large number of participants for a rare entity as PTHS, all diagnosed participants that were recruited through support groups, which led to a high rate of participation, the careful and secure way data are gathered and stored [14], building of the questionnaire with help of the patient support groups, physicians and social scientists, and the automated, cheap system of data gathering which facilitates long-term data gathering.

Weaknesses of the present study are that we relied on data from questionnaires, and did not perform a standardized physical assessment of participants ourselves, which is also a weakness as compared to other studies. With (ultra-)rare disorders in which in a single country the number of affected individuals is small, personal investigation of individuals is impossible for practical and financial reasons [16]. For several participants questions had a (markedly) retrospective character, which may have caused a recall bias. We will continue to include affected individuals in the future, in whom such recall bias is not present, which should allow us to compare answers by parents of young and older participants, and to determine whether this truly is a problem. Moreover clinical items may be expected to be easier to answer for parents than questions on electrophysiology or imaging. Still, families turn out to be well aware of results of studies performed in their child. We are planning to ask permission of participants to collect for instance the neuroimaging studies during follow-up.

There may also be an inclusion bias. We have no information about non-participating affected individuals. One may expect that parents with higher educational levels or those who are more active within support groups, are more likely to participate. This potential bias is present in many similar studies. We try to overcome this by making access to the study and filling out the questionnaire as simple as possible, and by including more participants with time. Furthermore we are working on translating the questionnaire in multiple other languages, allowing families to fill out a questionnaire using their mother tongue.

At follow-up we will add several standardised behaviour checklists to the waihonapedia system. We have gathered a series of 8 questionnaires, all validated and normalized, and which are at the present being translated in the same large number of languages as the physical questionnaire, in a scientifically acceptable manner. The repetitive follow-up will allow yielding valuable information on natural history of physical characteristics and behaviour of individuals with PTHS. This information can potentially also be used as historical normal values if interventions are planned.

## Conclusion

The web-based questionnaire system waihonapedia is an effective way to collect data on larger cohorts of people with rare diseases globally as it secures privacy, is fully automated and cheap. The first included cohort of 101 people with Pitt-Hopkins syndrome gives valuable information on health, behaviour and development. The number of participants will be expanded and there will be a repetitive follow-up. The total project should lead to better care of individuals with PTHS and similar disorders.
